# Nationwide cross-sectional survey of schistosomiasis and soil-transmitted helminthiasis in Sudan: study protocol

**DOI:** 10.1186/s12889-017-4719-4

**Published:** 2017-09-12

**Authors:** Seungman Cha, Sung-Tae Hong, Young-Ha Lee, Keon Hoon Lee, Dae Seong Cho, Jinmoo Lee, Jong-Yil Chai, Mousab Siddig Elhag, Soheir Gabralla Ahmad Khaled, Mustafa Khidir Mustafa Elnimeiri, Nahid Abdelgadeir Ali Siddig, Hana Abdelrazig, Sarah Awadelkareem, Azza Tag Eldin Elshafie, Hassan Ahmed Hassan Ahmed Ismail, Mutamad Amin

**Affiliations:** 1 Korea Association of Health Promotion, Gangseo-gu, Seoul, 07653 South Korea; 2 Faculty of Infectious and Tropical Disease, London School of Hygiene & Tropical Medicine, Keppel Street, London, WC1E 7HT UK; 3 Department of Parasitology and Tropical Medicine Seoul National University College of Medicine, Daehak-ro, Seoul, 03080 South Korea; 4 Department of Infection Biology, Chungnam National University School of Medicine, Daejeon, 35015 South Korea; 5 Community Interventions Division, Communicable and Non-Communicable Diseases Control Directorate, Federal Ministry of Health, Khartoum, Sudan; 6 Department of Preventive Medicine & Epidemiology Faculty of Medicine, Alneelain University, Khartoum, Sudan; 7 Data Management Unit Evidence Generation Department, Public Health Institute, Khartoum, Sudan; 8 Laboratory Coordination Unit, Case Management Department, Communicable & Non Communicable Diseases Control Directorate Federal Ministry of Health, Khartoum, Sudan; 9 Community Interventions Division and NTDs, Directorate for Communicable and Non-Communicable Diseases, General Directorate for Primary Health Care, Federal Ministry of Health, Khartoum, Sudan; 10 Research and Grants Unit, Ahfad University for Women, Omdurman, Khartoum, Sudan

**Keywords:** Nationwide survey, Schistosomiasis, Soil-transmitted helminthiasis, Mass drug administration, Mapping

## Abstract

**Background:**

Schistosomiasis and soil-transmitted helminthiasis (STHs) are target neglected tropical diseases (NTDs) of preventive chemotherapy, but the control and elimination of these diseases have been impeded due to resource constraints. Few reports have described study protocol to draw on when conducting a nationwide survey. We present a detailed methodological description of the integrated mapping of schistosomiasis and STHs on the basis of our experiences, hoping that this protocol can be applied to future surveys in similar settings. In addition to determining the ecological zones requiring mass drug administration interventions, we aim to provide precise estimates of the prevalence of these diseases.

**Methods:**

A school–based cross-sectional design will be applied for the nationwide survey across Sudan. The survey is designed to cover all districts in every state. We have divided each district into 3 different ecological zones depending on proximity to bodies of water. We will employ a probability-proportional-to-size sampling method for schools and systematic sampling for student selection to provide adequate data regarding the prevalence for schistosomiasis and STHs in Sudan at the state level. A total of 108,660 students will be selected from 1811 schools across Sudan. After the survey is completed, 391 ecological zones will be mapped out. To carry out the survey, 655 staff members were recruited. The feces and urine samples are microscopically examined by the Kato-Katz method and the sediment smears for helminth eggs respectively. For quality control, a minimum of 10% of the slides will be rechecked by the federal supervisors in each state and also 5% of the smears are validated again within one day by independent supervisors.

**Discussion:**

This nationwide mapping is expected to generate important epidemiological information and indicators about schistosomiasis and STHs that will be useful for monitoring and evaluating the control program. The mapping data will also be used for overviewing the status and policy formulation and updates to the control strategies. This paper, which describes a feasible and practical study protocol, is to be shared with the global health community, especially those who are planning to perform nationwide mapping of NTDs by feces or urine sampling.

**Electronic supplementary material:**

The online version of this article (10.1186/s12889-017-4719-4) contains supplementary material, which is available to authorized users.

## Background

Neglected tropical diseases (NTDs) affect an estimated 2.7 billion people, who are mainly poor, unreached, and belong to otherwise marginalized populations, in tropical and subtropical areas of the world [1]. Schistosomiasis and soil-transmitted helminthiasis (STH) are major target neglected tropical diseases (NTDs) of preventive chemotherapy, but the control and elimination of these diseases have been impeded due to resource constraints [2].

Schistosomiasis is a water-borne trematode infection that has been reported in 78 countries. It is most prevalent in sub-Saharan Africa, where more than 90% of those infected live. In the Eastern Mediterranean region, Yemen, Somalia, and Sudan remain as endemic countries [3]. It is reported that more than 218 million people required preventive chemotherapy for schistosomiasis in 2015 [4]. According to the World Health Organization (WHO), approximately 200,000 deaths take place due to schistosomiasis globally each year [4]. Women and children are an especially vulnerable group due to their frequent contact with contaminated water.

STHs, which include *Ascaris*, *Trichuris*, and hookworms transmitted by eggs present in human feces, are among the most common infections worldwide [5]. It is prevalent over 20% of population where sanitation is poor. Many infections occur in sub-Saharan Africa, the Americas, China, and Southeast Asia. Over 270 Million preschool aged children and over 600million school aged children need treatment and preventive interventions [5].

Although schistosomiasis and STHs are considered endemic in Sudan, with varying prevalence rates by state [6–8], there has never been a nationwide survey for these diseases, and even the prevalence rates reported in most states were not calculated on a solid epidemiological basis. Therefore, the Ministry of Health (MOH) was not able to develop an adequate strategy to control and eliminate these diseases in Sudan, including a plan for a nationwide mass drug administration (MDA) intervention.

MDA targeting the at-risk population is an essential public health effort for combatting NTDs [9]. In order to conduct a nationwide MDA program, it should be demonstrated whether a country has surpassed the threshold for intervention set by the World Health Organization (WHO) [10, 11] in certain administrative units.

Each NTD has its own methodology for mapping [10, 12, 13], but little is known about the details of nationwide surveys, such as the necessary scale of the workforce, logistics, and the like. Although large-scale mapping of schistosomiasis and/or STHs has been conducted in many countries [14–23], few reports have published on the study protocol for conducting a nationwide survey. This necessitates the publication of a feasible and practical study protocol to be shared with the global health community, especially those who are planning to perform a nationwide survey for these diseases, so that it can be adapted and applied with savings of cost and time.

We planned to conduct a nationwide survey for the Sudan-Korea schistosomiasis and STH elimination project (SUKO Project). The survey intended to identify the ecological zones requiring urgent MDA intervention for schistosomiasis and STHs across Sudan and to study the epidemiological and geographical pattern of these diseases in order to formulate control strategies. In this paper, we present a detailed methodological description of the integrated mapping of schistosomiasis and STHs on the basis of our experiences, hoping that it will be applied to future surveys in similar settings. In particular, this paper describes novel methodologies for the sampling frame and sampling method for a nationwide schistosomiasis and STHs survey to enhance epidemiological rigor while supporting field practicality. Adjusting the WHO guidelines [10], we have divided localities (equivalent to a district) into 3 different ecological zones depending on proximity to bodies of water based on the recommendation of a previous study [24] for more targeted surveys and interventions. In addition to determining whether an ecological zone has attained the threshold for MDA intervention, we aim to provide adequate prevalence estimates for schistosomiasis and STHs on the state level in Sudan by employing a probability-proportional-to-size (PPS) sampling method for schools and systematic sampling for student selection. In addition, morbidity information is essential to evaluate severity of disease, and we thus applied the abdominal ultrasound scanning [25].

## Methods/design

### Survey design/survey area

A school–based cross-sectional design was applied for the nationwide survey across Sudan as recommended by the WHO [10]. Sudan is the third largest country in Africa. The land is drained by the River Nile proper and its main tributaries, the Blue Nile, Atbara, al-Rahad, and al-Dindir from the Ethiopian plateau, and the White Nile from the lake region of Uganda and central Africa. Temperatures do not vary greatly with the season at any location; the most significant climatic variables are rainfall and the length of the dry season. Dust storms and periodic episodes of drought and flooding are not uncommon. The country faces soil erosion, desertification, and inadequate supplies of portable water, and its wildlife is threatened by excessive poaching. Climate change and the resulting increase in temperature may be causing tropical diseases and vectors to spread [26].

### Survey population

With an annual growth rate of 2.8%, the total population as projected from the 2008 census was 37.4 million people in 2016. Increasing urbanization has taken place, and natural disasters, civil conflicts, and poor conditions in rural areas have contributed to this demographic dislocation. Overall, 45.6% of the population is younger than 15 years of age, including 16.4% under 5 years. Over 50 % (50.5%) of the population is in the age group of 15–64 years, and 3.9% are 60 years of age or older. Life expectancy at birth is 59 years, and 83 of every 1000 children do not live to see their fifth birthday. Sixty-one percent of the population has access to an improved drinking water source, while 27% enjoy improved sanitation [27]. The target population is the whole Sudanese people but study population is Sudanese children aged 8–13 of 18 States.

### Sampling method

Sudan has 18 states and 189 localities, in which 15,761 schools exist. We have divided each locality into 3 different ecological zones depending on proximity to bodies of water (near, less than 1 km; medium, 1–5 km; far, 5 km or more) based on the guidance of state government officials. In this paper, we define an ecological zone as an area with a similar distance to bodies of water within a locality. We found that many localities have 1 or 2 ecological zones.

Two-stage cluster sampling was used for the nationwide survey. The first stage is to sample schools in each ecological zone, and the second stage is to sample students in a school. Five schools were selected from each ecological zone, and 60 students (30 boys and 30 girls) were sampled among the second-, fourth-, and sixth-grade students from each school. The WHO recommends 50 students per school [10], but we decided to sample 10 more students, considering a possible 16% non-response rate, which would mean 10 students not submitting their specimens.

**Fig. 1:**
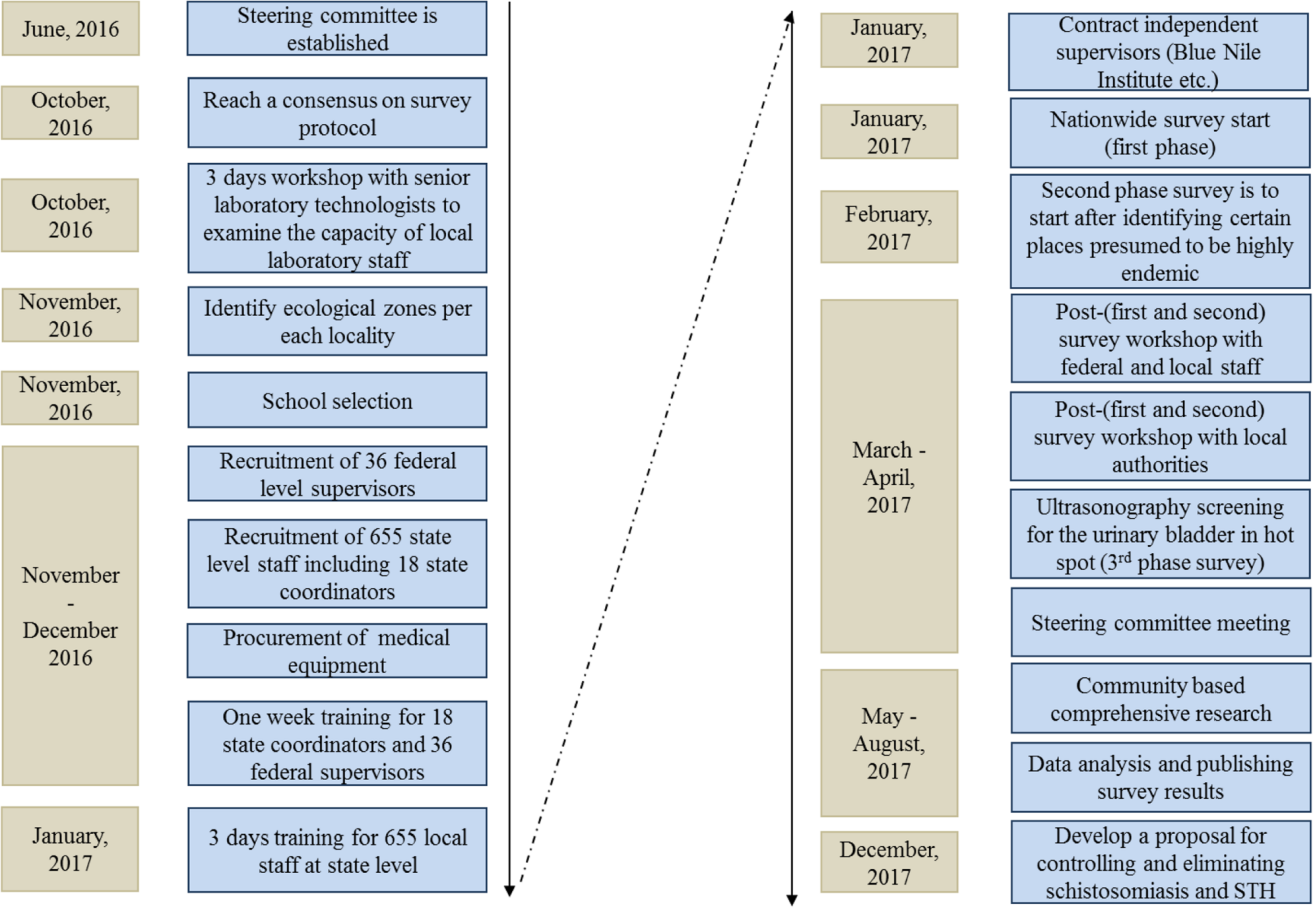
Flow diagram of nationwide survey of schistosomiasis and STH in Sudan

Schools were selected using the PPS method in each ecological zone. If a school is located in an insecure area, it was excluded. The recruitment of students continued until 20 children between the age of 8–13 years had been recruited using systematic sampling in each grade, starting with a number randomly chosen by a data collector. If a school did not have sufficient students in a grade, upper- or lower-grade classes were visited until specimens were collected from 60 students at the school (Fig. 1).

### Inclusion and exclusion criteria

In each school, children aged 8–13 years are eligible. Students were excluded from the study if they had not attended the same school for at least the past 6 months, or did not agree to be examined. Students were also excluded if they had been treated for schistosomiasis or STHs in the previous 6 months. Students with diarrhea were also excluded.

### Sample size

Ultimately, 108,660 students were selected from 1811 schools across Sudan. After the survey is completed, 391 ecological zones will be mapped out (Table 1).

**Table 1:** Number of schools and students for mapping by state

	State	No. of localities	No. of ecological zones	Number and proportion of schools						Total	Number and proportion of students				Total
				Boys		Girls		Mixed			Boys		Girls		
1	Blue Nile	7	15	27	(39.7)	17	(25.0)	24	(35.3)	68	2340	(57.4)	1740	(42.6)	4080
2	Gezira	8	22	28	(26.9)	30	(28.8)	46	(44.2)	104	3060	(49.0)	3180	(51.0)	6240
3	Central Darfur	9	23	11	(10.7)	12	(11.7)	80	(77.7)	103	3060	(49.5)	3120	(50.5)	6180
4	East Darfur	9	16	23	(30.3)	21	(27.6)	32	(42.1)	76	2340	(51.3)	2220	(48.7)	4560
5	Gadaref	12	21	35	(34.0)	38	(36.9)	30	(29.1)	103	3000	(48.5)	3180	(51.5)	6180
6	Khartoum	8	20	57	(57.0)	38	(38.0)	5	(5.0)	100	3570	(59.5)	2430	(40.5)	6000
7	North Kordofan	9	14	15	(24.2)	11	(17.7)	36	(58.1)	62	1980	(53.2)	1740	(46.8)	3720
8	North Sudan	7	14	21	(32.3)	18	(27.7)	26	(40.0)	65	2040	(52.3)	1860	(47.7)	3900
9	West Darfur	8	15	19	(26.4)	19	(26.4)	34	(47.2)	72	2160	(50.0)	2160	(50.0)	4320
10	West Kordofan	14	38	51	(27.3)	45	(24.1)	91	(48.7)	187	5790	(51.6)	5430	(48.4)	11,220
11	Kassala	11	12	22	(36.7)	18	(30.0)	20	(33.3)	60	1920	(53.3)	1680	(46.7)	3600
12	North Darfur	18	24	25	(21.7)	29	(25.2)	61	(53.0)	115	3330	(48.3)	3570	(51.7)	6900
13	Red Sea	10	15	14	(24.6)	13	(22.8)	30	(52.6)	57	1740	(50.9)	1680	(49.1)	3420
14	River Nile	7	16	18	(31.0)	12	(20.7)	28	(48.3)	58	1920	(55.2)	1560	(44.8)	3480
15	Sinnar	7	14	16	(31.4)	16	(31.4)	19	(37.3)	51	1530	(50.0)	1530	(50.0)	3060
16	South Darfur	20	51	41	(16.5)	44	(17.7)	164	(65.9)	249	7380	(49.4)	7560	(50.6)	14,940
17	South Kordofan	14	36	42	(26.3)	45	(28.1)	73	(45.6)	160	4710	(49.1)	4890	(50.9)	9600
18	White Nile	9	25	22	(18.2)	30	(24.8)	69	(57.0)	121	3390	(46.7)	3870	(53.3)	7260
Total		187	391	487	(26.9)	456	(25.2)	868	(47.9)	1811	55,260	(50.9)	53,400	(49.1)	108,660

**Required sample size for additional purposes** (precise estimation of schistosomiasis and STHs prevalence).

Although our main objective for nationwide mapping is to determine where MDA intervention is needed in each ecological zone, the sampling methodology we applied allows a precise estimation of schistosomiasis and STHs prevalence at the state level. Since the schools and students were randomly selected, we can estimate the prevalence of schistosomiasis and STHs at state level if the sample size is sufficient for the estimation.

Table 2 shows the required number of clusters and students to estimate prevalence using a priori guess for the parameters. The equation for calculating the sample size for a schistosomiasis prevalence survey, corrected for the design effect, is shown below: N = (1.96)^2^ × {(1 − p)/(d^2^ × p) × {1 + (m − 1) × k^2^× p/(1 − p)}.

**Table 2:** Sample size for precise estimate at state level using a priori guess

State	Presumed prevalence	Presumed k (the coefficient of between-cluster variation)	Precision	Cluster size	Sample size	Number of schools
State 1	7%	0.5	20%	60	2693	45
State 2	20%	0.5	20%	60	1801	30
State 3	5%	0.5	20%	60	3241	54
State 4	30%	0.5	20%	60	1641	27

For example, taking 5% as the population prevalence of schistosomiasis, 60 for the suggested cluster size, 0.5 as the between-cluster variation, and 20% precision, we would require a sample size of 3241 students from 54 schools. Based on very conservative estimations of parameters, prevalence, and cluster variation, the sample size we selected is sufficient to represent statewide prevalence. Therefore, we aim to map precise estimates of the prevalence at state level across Sudan using the same data for mapping the needs for MDA interventions at the ecological-zone level.

### Methods of stool and urine examination

Each student were asked to provide stool and urine samples. All samples were processed within 24 h. Fecal samples were examined for eggs of *S. mansoni* and STH using 2 smears with the Kato-Katz technique within 24 h of sampling. In addition to detecting *S. mansoni* eggs, we examined other eggs of intestinal helminths during the screening of the stool smears. Urine samples were tested for *S. haematobium* eggs by the centrifugation method using a centrifuge and microscope.

### List of survey variables for nationwide mapping

While collecting specimens from 108,660 students, a very concise questionnaire (Additional file 1: Appendix A) focusing on the essentials were administered to gather information on the following variables: demographic characteristics, such as age, gender, and parents’ occupation; sources of water at home; availability and types of latrines at home; defecation behavior (open defecation or not); water contact practices among the study population, such as bathing, washing, swimming, playing, and fishing; and the main reason for coming into contact with water.

### National coordinators

The Director of Community Health of the Federal Ministry of Health was designated as the chief national coordinator by the Steering Board. Under the chief national coordinator’s supervision, a group of national coordinators consisting of the officials of the Federal Ministry of Health was established, and they took on the responsibility of day-to-day supervision of the survey at the central level.

### Quality assurance

We employed internal and external quality assurance mechanisms to ensure that the laboratory work is of high quality. Eighteen teams of federal-level supervisors (federal laboratory supervisors and federal field supervisors) were established by the Federal Ministry of Health, comprising laboratory technicians and government officials for supervising data collection and laboratory examinations, and were deployed to each state during the entire survey period. For central-level quality control, a minimum of 10% (10,000–11,000 samples) of slides were rechecked by a federal laboratory supervisor, for which logbooks were in place to record the daily results of quality control. The federal supervisors provided technical input regarding the activities of the state coordinators.

Two senior professional technologists from the Blue Nile Institute and 3 professors from 3 different universities in Khartoum were contracted for external and independent quality control. They visited the laboratories on a state-by-state basis to validate the results by rechecking 5% of slides examined on the same day or the day before their visit.

### Data collectors, laboratory technicians, and state coordinators

The full survey team consists of laboratory technicians, laboratory assistants, interviewers, sample collectors, and cleaners. The number of the workforce ranges from 30 to 54 by state, comprising both genders and including individuals with adequate experience in school-based and community-based surveys, with a total number of 655. A minimum of 12 laboratory technicians and 4 laboratory assistants, and a maximum of 22 laboratory technicians and 7 laboratory assistants were recruited in each state depending on the sample size, and most of them are state-run hospital laboratory technicians and assistants. All of the laboratory technicians have a bachelor’s or a master’s degree in parasitology, laboratory science, or a similar subject, and the laboratory assistants have a diploma in the same area. For collecting data, a minimum of 6 interviewers and 6 sample collectors, and a maximum of 12 interviewers and 12 sample collectors were recruited for administering the questionnaire and collecting stool and urine samples from students. Most of them are government officials of the State Ministry of Health, and their education levels are similar to those of the laboratory technicians. We ensured that the recruitment of staffs would not be delayed until close to the start of the survey to obtain ample time to train all staffs before implementing the survey. For each state, a state coordinator was assigned for state-level supervision. State coordinators have the task of ensuring the proper implementation and management of the survey activities, and report directly to the national coordinators. The state coordinators are qualified personnel with adequate experience in the conduct of research and surveys. The required number of laboratory technicians was calculated based on the assumption that 1 technician could examine 75 slides per day.

We ensured that every state team have at least 3 4-wheel-drive vehicles. The project team hired 36 rental cars to support the state teams and requested the Ministry of Health of every state to provide at least 1 vehicle to facilitate the survey. One data collector and 1 interviewer comprised 1 unit, and 2 units used 1 vehicle. One unit visited 1 school per day to interview 60 students and collect 60 urine and stool samples.

### Training set-up

Supervisors’ training: we arranged a 1-week training workshop for state coordinators, federal laboratory supervisors, and federal field supervisors from December 18, 2016 to December 22, 2016. The first 3 days were for all participants, during which the details of the survey protocol, questionnaires, and ethical issues were presented, and action plans were developed through group practices by state. The next 2 days were dedicated to federal laboratory technicians and federal field supervisors of laboratory examinations, and data collection and interviewing practices with a tablet PC. During the training, we investigated the validity of the procedure by rechecking the examination results by trainers.

The training of staffs is important to ensure that procedures are properly conducted and standardized throughout the survey, under all specific survey conditions. Therefore, we made every effort to train all the staff members systematically and assess their suitability for their posts. A booklet containing the study protocol, questionnaire, manual, and bench aids was developed and disseminated in all states. In addition, a poster containing essential information for laboratory examinations for schistosomiasis and STHs was produced and delivered.

### Federal-level training for state coordinators and federal supervisors

A training workshop was organized to train 18 state coordinators and 36 federal-level supervisors. The duration of the training was 3 days (24 contact hours). The training covered the following areas: objectives of the survey, obtaining informed consent (Additional file 2: Appendix B) from each participant, communication skills, interviewing techniques, filling in the questionnaire, data cleaning, collection and transport of specimens, and laboratory practices.

### Training for data collectors

All the laboratory technicians and data collectors (interviewers and sample collectors) were gathered together to be trained at the state level for 3 days in early January 2017. Detailed protocols for the school-based survey were explained to the data collectors, with a focus on the methods of student selection, administering the questionnaire, and collecting specimens.

### Training for laboratory technicians

Lab technicians were trained at 2 levels. Thirty-six senior laboratory technologists at the state level were trained at the Blue Nile Institute in Gezira for 3 days (October 26–28, 2016) with a focus on the methods of examining urine and stool samples (centrifugation, filtration, dipstick, circulating cathodic antigen testing). During the training at the Blue Nile Institute, we conducted a field exercise at 2 schools to help to build capacity, particularly regarding student selection, questionnaire administration, and stool and urine sample collection within the survey team. A total of 254 laboratory technologists were trained at the level of each state for 3 days by federal laboratory supervisors during early January 2017. Standard laboratory techniques were used to conduct the required investigations.

### Laboratory settings

Central laboratories were established in each state, with a minimum of 3 centrifuges and 10 microscopes. For villages further than 1 day’s driving distance, mobile laboratories were set up, in which 1 centrifuge and 3 microscopes were installed.

### Site visits

Site visits were conducted with the purpose of interacting with stakeholders and making precise assessments of preparedness for mapping, especially regarding the amount of functioning medical equipment and utilities, the capability of the workforce according to position, and vehicle availability in late November 2016.

### Pre-testing and field training

In addition, before starting data collection, the skills of all laboratory technicians were examined at the state level by federal-level supervisors. For each team, pilot field training was conducted before starting to collect samples from selected schools. All the teams started sampling when they were assessed to be qualified based on re-examination and validation by the federal-level supervisors.

### Urine for *S. haematobium* eggs

Coded containers were used to collect urine specimens from the sampled students. The collected specimens were examined by the laboratory technicians on the same day in the central laboratory or in a field laboratory. Urine specimens were examined using the centrifugation method with a centrifuge and microscope. A centrifuge tube containing 10 mL of urine was placed in a table centrifuge (PLC-03, Gemmy Ind. Co., Taiwan) fitted with a 15 mL centrifuge tube head, and spun at 2000 rpm for 15 min. The supernatant fluid was decanted, and the sediment was examined under a 10 X objective microscope.

### Stool for *S. mansoni* eggs

Stool specimens were examined for eggs of *S. mansoni* or STHs using 2 smears of the Kato-Katz method. The Kato-Katz method was implemented following the WHO guideline of stool examination [28]. The stool smears were prepared using plastic templates (MH Healthcare Co., Seoul Korea) which was qualified by the Korean Association of Health Promotion.

### Ethical considerations

We have obtained ethical review and approval both from the Institutional Review Board of Federal Ministry of Health, Sudan (FMOH/DGP/RD/TC/2016; January 15, 2017) and the Korea Association of Health Promotion (130750–20,164-HR-020; May 16, 2016). All the Ministries of Health at the state level were provided with the survey protocol and a description of the proposed activities, and approval for the survey was granted prior to implementation.

After selecting target schools, all the head teachers were gathered together at the state level and they were trained on the study protocol by state coordinators. In particular, they were trained that they have to inform parents of this study, in particular the key elements of the study reflecting the informed consent form, and parents must be given the option to opt-out their child from the participating in the study. The head teachers trained teachers in his/her school as they were trained. Teachers educated their students for all the details, and ask them to inform their parents before survey team would visit the school. If some parents did not agree on their child’s participation, the child was excluded.

The purpose and details of the survey were explained to head teacher of each school before he or she would be requested to provide informed consent in written format for the students to participate in the survey.

If consent is granted, the details of the survey were explained to each student who met the inclusion criteria. A separate form of informed consent for students was developed and the script was read by data collectors and every detail was explained to students point by point (Additional file 2: Appendix B). The children were then asked whether they would participate in the survey. The name of each participant providing consent was documented to record verbal consent. We considered it very difficult to obtain written consent from the parents of school children due to the large sample size, and we obtained approval on this procedure from the Institutional Review Board of Federal Ministry of Health, Sudan. The protocol of this survey for informed consent complies with the standard procedure of Federal Ministry of Health, Sudan.

Only those who provide informed consent were requested to provide urine and stool samples. Students who tested positive for schistosomiasis or STHs were immediately provided with a treatment dose of praziquantel or albendazole, respectively, following WHO recommendations [10] and the regulations of the Federal Ministry of Health, Sudan [6].

### Additional survey after finding areas suspected to be highly endemic (survey phase 2)

**Fig. 2:**
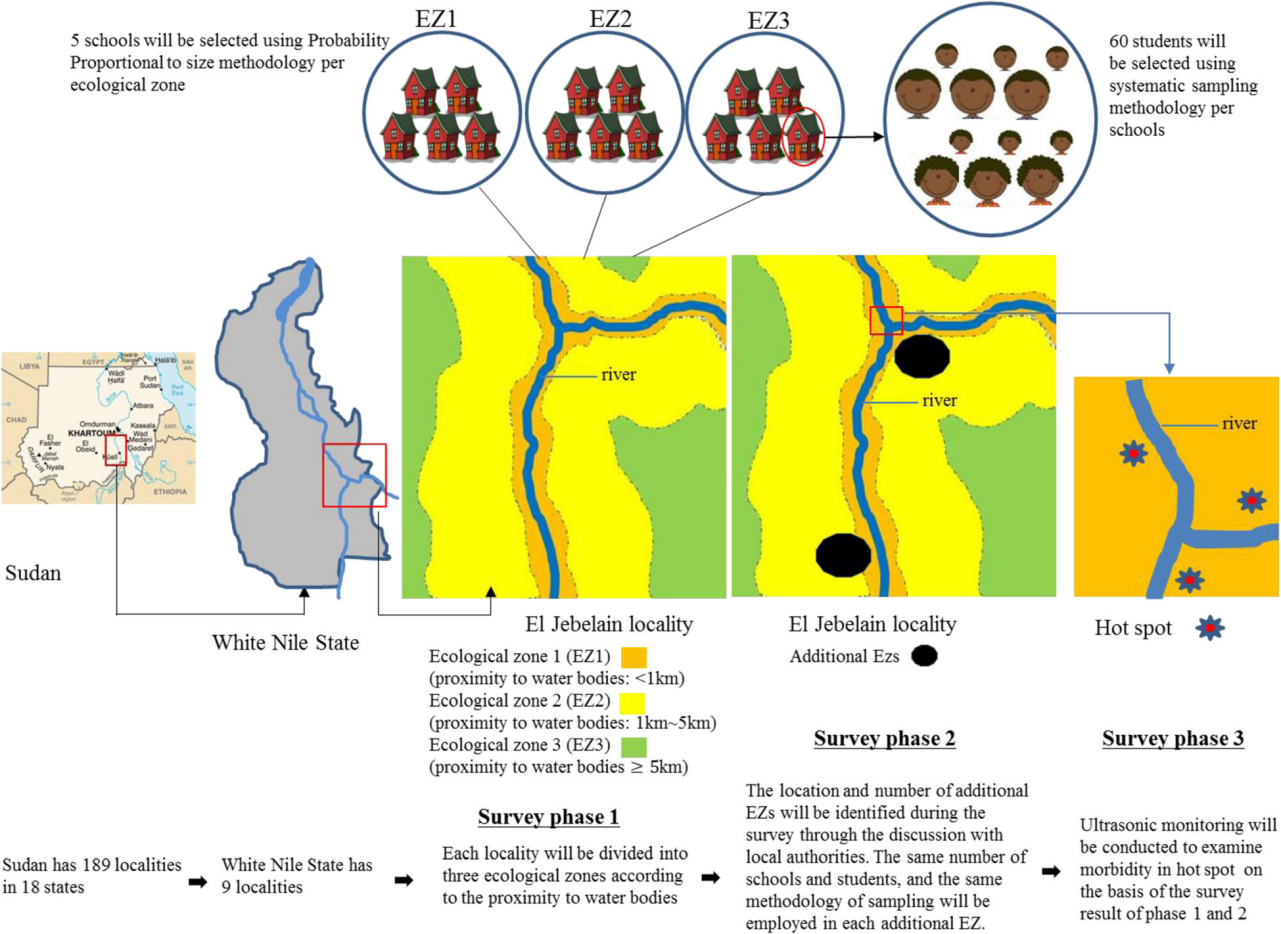
Sampling methods of school and students selection in each survey phase

During the survey period, we attempted to identify any unselected areas suspected to be highly endemic through in-depth discussions with local health workers and community members, and then an additional survey will be conducted targeting the specific area (survey phase 2) as shown in Fig. 2.

### Sentinel sites

Hot spots in each state will be listed, and some of them will become sentinel sites for monitoring and evaluation after MDA. (survey phase 3).

### Ultrasonography monitoring

Ultrasonography (US) screening of the urinary bladder (UB) will be applied to a selected subset of the survey target population in order to monitor the morbidity of the UB. The use of US may compensate the low diagnostic sensitivity of urine microscopy. US may be implemented for at least 500 individuals in the sentinel sites of each state. US is recommended after the baseline survey and before MDA [29]. The US procedure will follow the modified WHO guidelines [30]. Persons who are assigned to carry out US will be trained to implement the appropriate diagnostic criteria. To monitor the urinary bladder morbidity, ultrasound scanning of the lower abdomen is applied using the ultrasound scanner (Voluson-e, General Electric Co., WI). The images will be interpreted following the modified Niamey criteria [30].

### Steering board

The Steering Board is ultimately responsible for designing the study, maintaining the quality of the conduct of the study, and writing the final study report. The Steering Board comprises 8 members who are Sudanese and Korean parasitologists or health specialists as well as officials of the Sudanese government and the WHO. It will monitor and intervene as appropriate throughout the entire process of survey design, implementation, and analysis.

### Plan for data analysis

Tablet PCs (SM-Galaxy T350NZAAXAR/Samsung/Seoul, Korea; MediaPad T1 7.0/Huawei/ Shenzhen, China) were used to administer the questionnaires, and the completeness, correctness, and consistency of answers were checked on the spot. The laboratory results were entered into the tablet PCs by state coordinators. All the data were submitted to the central level by state coordinators, and national coordinators analyzed the progress of the survey and survey results on a daily basis. Using the tablet PCs, we could determine the longitude and latitude of a school, but this was difficult if the tablet PC was not connected to the internet. In such cases, a handheld GPS device (eTrex, Garmin International, Olathe, KS, USA) was used to collect the coordinates of the schools.

The Statistical Package for Social Sciences (SPSS) version 21 (IBM Corp., Armonk, NY, USA) will be used for the data analysis. Maps of schistosomiasis and STH prevalence will be developed using ArcGIS version 9.2 (Esri, Redlands, CA, USA).

## Discussion

This nationwide survey is expected to generate important epidemiological information and indicators about schistosomiasis and NTDs that will help us in monitoring and evaluating the control program. Nationwide survey indicators are also useful for visualizing the overall situation of the country, which can facilitate further policy formulation and updates of control strategies. The information generated by this survey can be used by the program staff and other concerned bodies to advocate for policy changes and the mobilization of extra resources in support of control efforts across the country. The survey results can be used to identify priority areas for further operational research and exploration.

In many previous studies [14–23], villages or schools were selected among those considered highly endemic by local health workers. However, we are not sure whether they were always well-informed about where schistosomiasis was prevalent. To overcome this limitation, we employed 2 novel methodologies. First, we divided each district into 3 ecological zones (i.e., high, medium, and low transmission risk of schistosomiasis). Second, during the survey period (survey phase 2), we will identify any unselected areas suspected to be highly endemic through in-depth discussions with local health workers and community members, and then an additional survey will be conducted targeting the specific area.

Pelletreau and colleagues [24] recommended sub-districts as the mapping and implementation units for MDA because district-based surveys may underestimate or overestimate the prevalence of schistosomiasis. This study, by incorporating their recommendations, will produce more rigorous results and contribute to more cost-efficient MDA interventions by generating more targeted results.

Additionally, this study will allow a precise estimate of schistosomiasis and STH prevalence at the state level because schools and students were randomly selected according to the PPS and systematic sampling respectively, which may overcome the limitations of previous studies [14–23] that only aimed to guide MDA interventions. Thus, the results of this survey will be used as a baseline for measuring the effects of MDA and/or other health interventions at the state level.

The WHO recommends a series of intervention thresholds for the diseases to be mapped. In Sudan, since we plan to estimate the prevalence in each ecological zone designed for this mapping project, MDA will be conducted across zones demonstrating a schistosomiasis and STH prevalence of over 10% [10]. Communities where schistosomiasis is endemic or hyperendemic will be treated with praziquantel every other year or annually, and communities where STH is endemic or hyperendemic will be treated with albendzole on an annual or biannual schedule. We assume that 3.7 million people (10% of the Sudanese population) will benefit from MDA for schistosomiasis along with the nationwide mapping.

We have been employing robust way of quality assurance. We expect the validity of the survey to be highly ensured by having 10% of samples reexamined by central level supervisors and 5% by independent supervisors.

The main objective of nationwide mapping is to find ecological zones requiring MDA intervention, and we therefore have not administered a comprehensive questionnaire to all students to identify the epidemiological characteristics and risk factors. We thus propose a separate community-based survey, distinct from the nationwide mapping, to identify key findings related to epidemiological features and risk factors. From a statistical point of view, 108,660 samples are a waste of resources and not necessary for an investigation of risk factors through a comprehensive household-based questionnaire. A separate survey protocol will be developed for a community-based survey with a comprehensive questionnaire to investigate the epidemiological features and risk factors of schistosomiasis and STHs.

There have been some challenges or limitations of this survey. Sudan has some states politically insecure such as Darfur state, where it took longer than in other states. There were some schools located far from the capital city of each State. Although we have run mobile laboratory for getting to the schools located at remote villages, we are still concerned about the logistical issues for those in hard-to-reach area. Strong political and logistical support of State government is critically important for successful implementation in the area.

## Additional files

Additional file 1: Appendix A. Questionnaire for subjected students. (DOCX 26 kb)

Additional file 2: Appendix B. Informed consent forms for students and teachers. (DOCX 15 kb)

## Abbreviations

GPS: global positioning system
MDA: mass drug administration
MOH: Ministry of Health
NTDs: Neglected tropical diseases
PCs: personal computers
PPS: probability-proportional-to-size
SPSS: Statistical Package for Social Sciences
STH: soil-transmitted helminthiasis
WHO: World Health Organization
