# Supplementary material for: Nationwide cross-sectional survey of schistosomiasis and soil-transmitted helminthiasis in Sudan: study protocol
Source: BMC Public Health. 2017 Sep 12;17:703. doi: 10.1186/s12889-017-4719-4 (PMC5596840; doi:10.1186/s12889-017-4719-4)
Supplement: Supplementary file 1 — Appendix A. Questionnaire for subjected students. (DOCX 26 kb) [file 12889_2017_4719_MOESM1_ESM.docx]

**Appendix A**

***Questionnaire for students***

Name_________________

State Locality Ecological Zone School Student

1. Age [ ]
2. Sex ① boy ② girl
3. Parents’ occupation [ ]
4. What types of water source do you for drinking at home?
5. protected (boreholes, hand-pumps, protected spring)
6. unprotected (river, stream, pond, unprotected spring)
7. Do you contact water bodies (river, stream, lake, irrigation canal, reservoir) more than two times a week?
8. Yes ② No
9. Why do you contact water bodies?
10. Fetching water ② bathing ③ laundry ③ playing (swimming) ④ for livestock
11. Others(________________)
12. Do you have latrine in your household compound?
13. I don’t have any latrine ② Simple pit latrine
14. Ventilated improved pit latrine ④ flush toilets
15. Where do you defecate? ① Latrine ② open defecate

***Observation for a school ( questionnaire for head teachers)***

Name

State Locality Ecological Zone School

1. Geographic location of the school: [observation]
2. What types of water source does the school have? [observation]
3. protected (boreholes, hand-pumps, protected spring)
4. unprotected (river, stream, pond, unprotected spring)
5. Does the school have latrine school compound? [observation]
6. It doesn’t have any latrine ② Simple pit latrine
7. Ventilated improved pit latrine ④ flush toilets
8. Please describe all the surrounding villages where students live.
9. Does the school have any curriculum to regularly teach Bilharzia and STH?

①Yes ② No

1. Has the school had any special health program, campaign such as nutrition, vaccination or any other? Please describe it if there has been.

_________________________________________________________

1. When was last time when MDA intervention was conducted?

(Month: Year: )
