# Supplementary material for: Nationwide cross-sectional survey of schistosomiasis and soil-transmitted helminthiasis in Sudan: study protocol
Source: BMC Public Health. 2017 Sep 12;17:703. doi: 10.1186/s12889-017-4719-4 (PMC5596840; doi:10.1186/s12889-017-4719-4)
Supplement: Supplementary file 2 — Appendix B. Informed consent forms for students and teachers. (DOCX 15 kb) [file 12889_2017_4719_MOESM2_ESM.docx]

**Appendix B**

**Informed consent form for students (**to be read by data collectors)

I am ___________currently working as a data collector for nationwide mapping, which is being conducted under the guidance of Ministry of Health, Sudan. We are conducting this survey to determine whether an area surrounding your school needs treatment. After collecting urine and stools from you, we will examine the prevalence of schistosomiasis and STH, and we will determine whether the area including your school needs treatment. If the prevalence proved to be higher, drugs will be provided to all the people in the area including all the students in your school. If the prevalence is too low, drugs will be provided only to the students with positive diagnosis. In addition, I would like to ask you about water and sanitation conditions in your house and community. I expect to need approximately 20 -30 minutes of your time. If you agree to participate, the information you give me will be used only in anonymous form. Interview and diagnosis results will be treated as confidential and destroyed at the completion of the study. Any details which might identify you will not be shared. You should feel free not to provide any information you do not wish to share with me or to end the interview at any time. Do you have any questions about the purpose or process? Is there anything else you would like me to clarify? If you have any concerns about your treatment, you can contact your teacher, headmaster or ___________, state coordinator (phone number: ).

Thank you for your cooperation!

**Informed Consent (**to be read by students)

I have been explained on all the details about the survey including the objectives and specimens that I have to provide, if I agree. I have been explained that I can stop answering the questionnaire at any time I want and I can decide whether I provide my stools and urine by myself without any coercion. I consent to participating in this study.

School__________/Name__________/Date_____________

**Informed Consent for head teacher**

Hello, my name is ___________________________.

I am currently working as a data collector for nationwide mapping, which is being conducted under the guidance of Ministry of Health, Sudan. We are conducting this survey to determine MDA intervention targeting an area surrounding this school. As you know, Sudan has enormous burden of schistosomiasis and STH, however there is considerable uncertainty about the number of people infected with schistosomiasis and STH, which hinders us to deliver required medicine to the people who need treatment desperately. After collecting specimens, urine and stools from your students, we will examine the prevalence of schistosomiasis and STH, and we will determine whether the area including your school needs MDA intervention. If the prevalence proved to be higher, praziquantel and albendazole will be provided to all the people in the area including all the students in your school. If the prevalence is too low, drugs will be provided only to the students with positive diagnosis. In addition, we will administer questionnaires about water and sanitation conditions in your students’ house and community. Your cooperation will substantially contribute to effective distribution of essential medicines to the people with the urgent needs of treatment in your school and villages. The information the students give me will be used only in anonymous form. Interview and diagnosis results will be treated as confidential. Any details which might identify you will not be shared. The students should feel free not to provide any information they do not wish to share with me or to end the interview at any time. Do you have any questions about the purpose or process? Is there anything else you would like me to clarify? Thank you for your cooperation!

**Informed Consent (**to be signed by head teacher)

I have been informed on all the details about the survey including the objectives and specimens that students have to provide, if they agree. I have been explained that students can stop answering the questionnaire at any time they want and they can decide whether they provide their stools and urine by themselves without any coercion. I consent that the school participates in this study but every students has the rights to decide to participate or not.

School__________/Name_____________(sign)_____________/Date_____________
